# Uncovering Anticancer Mechanisms of Spiramycin Derivatives Using Transcriptomic and Metabolomic Analyses

**DOI:** 10.3390/metabo15100647

**Published:** 2025-09-27

**Authors:** Renyu Yang, Wuxiyar Otkur, Tingze Feng, Yirong Li, Shaojun Pei, Huan Qi, Yaopeng Zhao, Yao Lu, Hailong Piao

**Affiliations:** 1 State Key Laboratory of Phytochemistry and Natural Medicines, Dalian Institute of Chemical Physics, Chinese Academy of Sciences, Dalian 116023, China; 2 University of Chinese Academy of Sciences, Beijing 100049, China; 3 School of Life Science and Biopharmaceutics, Shenyang Pharmaceutical University, Shengyang 110016, China

**Keywords:** carrimycin, anticancer, metabolomics, transcriptomics

## Abstract

**Background**: Carrimycin is a mixture of spiramycin derivatives with antibacterial functions. However, recent studies have shown that it possesses certain anticancer properties. The specific mechanism of the anticancer activity is unknown. **Methods**: To study the anticancer mechanism of carrimycin, we synthesized a derivative of spiramycin, n-hexyl spiramycin (h-SPM), and used a combination of metabolomics and transcriptomics methods. Capillary electrophoresis–mass spectrometry (CE-MS) was used to detect polar small molecule metabolites, and liquid chromatography–mass spectrometry (LC-MS) was used to detect lipid metabolites in cells. Transcriptomics was used to measure mRNA content in cells. Finally, by processing these data using specific bioinformatics methods, the mechanism underlying anticancer effect of carrimycin was determined. **Results**: Metabolomics and transcriptomic results showed that lipid metabolism and mitochondrial biogenesis pathways in the cells changed after hSPM treatment, NR1D1 genes and ceramide were enriched from these pathways, implicating the involvement of ROS and pro-inflammatory response. Western blotting verified that the protein levels of NR1D1 decreased after h-SPM treatment, and ROS stating and qPCR demonstrated that ROS levels and the mRNA levels of pro-inflammatory genes were greatly induced by h-SPM. **Conclusions**: h-SPM reduced the protein level of NR1D1, disrupted metabolic regulation, accumulating ceramide, and the subsequent increased ROS generation promoted apoptosis and pro-inflammatory-like response of cells. Our findings unveiled the anticancer mechanism of a potent anticancer derivative of spiramycin and unveiled its mechanism of action.

## 1. Introduction

Carrimycin is a modified 16-membered macrolide antibiotic produced by recombinant Streptomyces strains. Its primary active components are several monomeric isovalerylspiramycins (ISPs) [1,2,3]. Beyond its established antibacterial properties, ongoing research has revealed additional pharmacological effects of ISPs, including anti-inflammatory activity and modulation of immune responses [4,5,6]. Given that tumor cells are characterized by high metabolic and proliferative activity, and that certain cancers are closely associated with chronic inflammation, ISPs present promising therapeutic potential in oncology [7]. Specifically, we focused on triple-negative breast cancer (TNBC) and glioblastoma, which are aggressive malignancies with limited treatment options and poor prognosis. TNBC, characterized by the absence of estrogen, progesterone, and HER2 receptors, lacks targeted therapies and remains a critical unmet clinical need. Similarly, glioblastoma is the most common and aggressive primary brain tumor, with high recurrence rates and dismal survival despite standard treatment.

**Figure 1:**
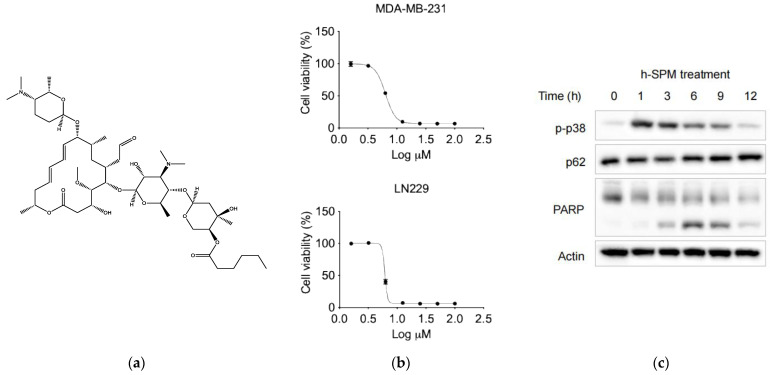
The basic properties of drugs. (**a**) The chemical structure of h-SPM; (**b**) Cell viability of MDA-MB-231 breast cancer cells and LN229 fibrous astrocytes treated with indicated concentrations of h-SPM, assessed using the CCK-8 assay; (**c**) Protein levels of p-p38, p62, and cleaved PARP in MDA-MB-231 cells analyzed by Western blot.

Notably, carrimycin has demonstrated inhibitory effects against liver and lung cancers in preclinical studies, suggesting its potential as an anticancer agent [8,9], but its effect on TNBC and glioblastoma has not been tested yet. In addition, due to its complex composition, elucidating its anticancer mechanisms remains challenging. During synthesis, specific functional groups in carrimycin undergo structural modifications, yielding derivatives with enhanced pharmacological activity. Among these, spiramycin derivatives have demonstrated notable anticancer potential. Here, we synthesized a novel derivative—h-SPM—as a representative compound for mechanistic investigation using models of TNBC (MDA-MB-231) and glioblastoma (LN229), which represent high-unmet-need malignancies (Figure 1a).

Multi-omics approaches offer powerful tools for elucidating drug mechanisms [10,11,12]. To comprehensively characterize the anticancer activity of h-SPM, we employed both metabolomic and transcriptomic analyses. For metabolomics, two complementary platforms were used: CE-MS to detect changes in polar small-molecule metabolites [13,14,15], and LC-MS for profiling non-polar lipid metabolites [16,17]. In parallel, transcriptomic analysis was conducted to examine h-SPM-induced changes in mRNA expression [18,19].

Subsequent data integration and pathway analysis were performed using bioinformatics tools to identify cellular pathways affected by h-SPM treatment [20,21,22]. We cross-referenced metabolic and gene expression changes with public databases to prioritize altered pathways [23,24], and selected high-frequency, functionally relevant genes within these pathways. Finally, we validated candidate target genes using Western blot and qPCR, confirming their potential roles in the anticancer activity of h-SPM.

## 2. Materials and Methods

### 2.1. Cell Lines and Cell Culture

MDA-MB-231 and LN229 cells were maintained in Dulbecco’s Modified Eagle Medium (DMEM) medium (Meilunbio) supplemented with 10% fetal bovine serum (FBS) (Multicell) and 1% penicillin–streptomycin (100 µ/mL penicillin and 100 μg/mL streptomycin) (Meilunbio). Cells were cultured in an incubator at the condition of 37 °C and 5% CO_2_.

### 2.2. Cell Counting Kit-8 (CCK8) Assays

MDA-MB-231 and LN229 cells were seeded at a density of 1 × 104 cells per well in 96 well plates. After 24 h, h-SPM were added to the medium at concentrations ranging from 0 to 100 μM. The medium with h-SPM was added 100 μL per well. After 48 h of treatment the medium was discarded and added the medium with 10% CCK8 reagent (Meilunbio). The cells were cultured for another 2 h, while the plates were analyzed by Cytation-5 at the wavelength of 450 nm. The cell viabilities were calculated by the absorbance.

### 2.3. Western Blotting

Cells were lysed using RIPA buffer, supplemented with protease and phosphatase inhibitors to prevent protein degradation. The protein concentration in the lysate was determined using a BCA kit (Beyotime, Shanghai, China). To equalize the protein concentration across samples, RIPA buffer was added to each lysate accordingly. The proteins were separated via 10% sodium dodecyl sulfate–polyacrylamide gel electrophoresis (SDS-PAGE) and then transferred onto PVDF membranes (Millipore Billerica, MA, USA). The membranes were incubated with primary antibodies at 4 °C overnight, followed by incubation with secondary antibodies for 1 h at room temperature. The protein bands were detected using chemiluminescent reagents and visualized with the Tanon-5200 chemiluminescent imaging system.

### 2.4. RNA Sequencing

Cells were seeded in 6 cm dishes. When the cell density reached approximately 80% confluence, the culture medium was replaced with medium containing the h-SPM. After 12 h of treatment, the medium was removed, and the cells were flash-frozen to preserve RNA integrity. TRIzol reagent (Takara, Shiga, Japan) was used to extract total RNA from the cells. The RNA concentration was measured using Cytation 5, and RNA quality was assessed by gel electrophoresis. RNA samples with two clear bands (indicative of intact ribosomal RNA) were deemed suitable for sequencing and were subsequently sent to a commercial company for RNA sequencing analysis. We used Illumina NovaSeq 6000 (Illumina, San Diego, CA, USA) for RNA sequencing. After the sequencing process is completed, the clusterProfiler 4.0 software is used for pathway enrichment analysis. The KEGG and Reactome databases are, respectively, employed for different analyses to obtain results from different perspectives.

### 2.5. LC-MS-Based Metabolomics Analyses

The experimental groups were the same as those used for RNA sequencing, but each group now had six replicate samples. For the lipid extraction process, a mixture of methanol and chloroform was used. Ultrapure water was then added to the mixture, and the extraction was centrifuged at 15,000× *g* for 15 min at 4 °C. The aqueous phase was carefully collected and freeze-dried in a vacuum concentrator to remove residual solvents. Following this, the samples were dissolved in the liquid phase of the chromatographic separation system. The separation was performed using an ACQUITY™ Ultra Performance Liquid Chromatography (UPLC) system (Waters, Milford, MA, USA). Finally, a coupled AB Sciex tripleTOF 5600 plus mass spectrometer (AB Sciex, Marlborough, MA, USA) was employed for global metabolomics and lipidomics profiling to identify and quantify the lipids and metabolites. The metabolomics analysis was conducted using the website metaboanalyst (https://www.metaboanalyst.ca/ (accessed on 13 December 2024)), and the enrichment pathway database was Homo sapiens (KEGG). The analysis of differential metabolites in metabolomics was performed using the Wilcoxon test statistical method.

### 2.6. CE-MS-Based Metabolomics Analysis

The same groups were set as the lipidomics analyses. When the medium was removed, the cells were washed with 5% mannitol once. Then the metabolomics were extracted by methanol. The analysis of the metabolites was performed by a CE system (G7100A, Agilent, Santa Clara, CA, USA) combing with time of flight (TOF) mass spectrometry (G6224A, Agilent). All samples were separated by the fused silica capillary [i.d. of 50 μm; total length of 80 cm; Human Metabolome Technologies (HMT), Tsuruoka, Japan]. Quantitative Analysis Software 10.2 (Agilent) was used for peak extraction and identification. The data processing method is the same as LC-MS.

### 2.7. qPCR Assay

Cells were cultured with h-SPM at a concentration of 10 μM for different time points (0, 3, 9, and 12 h). RNA was extracted from the cells using TRIzol reagent (Takara, Shiga, Japan). The extracted RNA was then divided into several PCR tubes. Each tube was mixed with SYBR Green and Primer Mix, and the tubes were placed on the qPCR instrument to obtain the expression level of each gene.

### 2.8. Reactive Oxygen Species (ROS) Determination

Cells were cultured in non-transparent 96-well plates following the same protocol as the CCK8 assay. The cells were treated with h-SPM at concentrations of 0 and 10 μM. After treatment, 100 μL of medium containing ROS reagent (1:2000, *v*/*v*) (Beyotime) was added to each well, and the cells were incubated for an additional hour. Following this incubation, the medium was removed, and the cells were washed three times with PBS. Finally, 100 μL of PBS was added to each well, and the plates were analyzed using the Cytation 5 in fluorescence mode at wavelengths of 485 nm (excitation) and 520 nm (emission). The ROS intensity was measured based on the data obtained.

### 2.9. Statistical Analysis

All the statistics of the experiments were represented as the mean ± standard deviation (SD) from three times of replicative experiments. Microsoft Excel 2019 and GraphPad Prism 6.04 were used to draw the charts. The *p*-value of the two groups was compared using Student’s *t*-test and the one-way analysis of variance using more than two groups. A value of *p* < 0.05 was used to indicate a statistically significant difference.

## 3. Results

### 3.1. Evaluation on the Anticancer Activity of h-SPM

To assess the anticancer efficacy of h-SPM, cell viability assays were performed using MDA-MB-231 and LN229 cancer cell lines. The results demonstrated that h-SPM exhibited significant cytotoxicity in both cell types (Figure 1b). When cells were treated with increasing concentrations of h-SPM (3–10 μM) for 48 h, a marked reduction in cell viability was observed, indicating its potent anticancer activity even at low micromolar levels. Based on these results, 10 μM was selected as the optimal concentration for subsequent mechanistic studies. Western blot analysis revealed that h-SPM treatment induced phosphorylation of p38 and cleavage of PARP (Figure 1c), markers associated with cell cycle arrest and apoptosis [25,26]. These findings confirm that h-SPM promotes cancer cell death, at least in part, through the induction of apoptosis.

### 3.2. Transcriptomic Profiling Reveals Involvement of Amino Acid, Lipid, and Mitochondrial Metabolism in h-SPM Action

**Figure 2:**
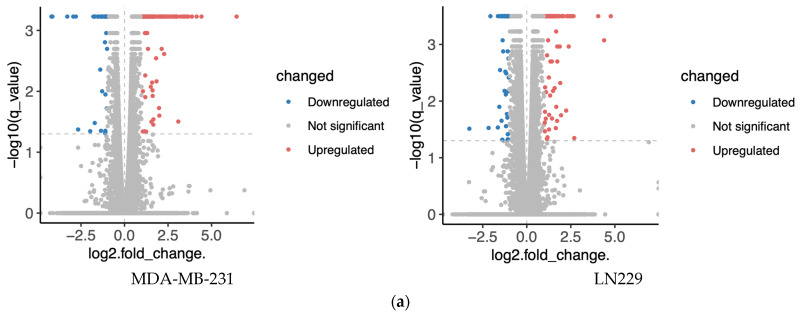

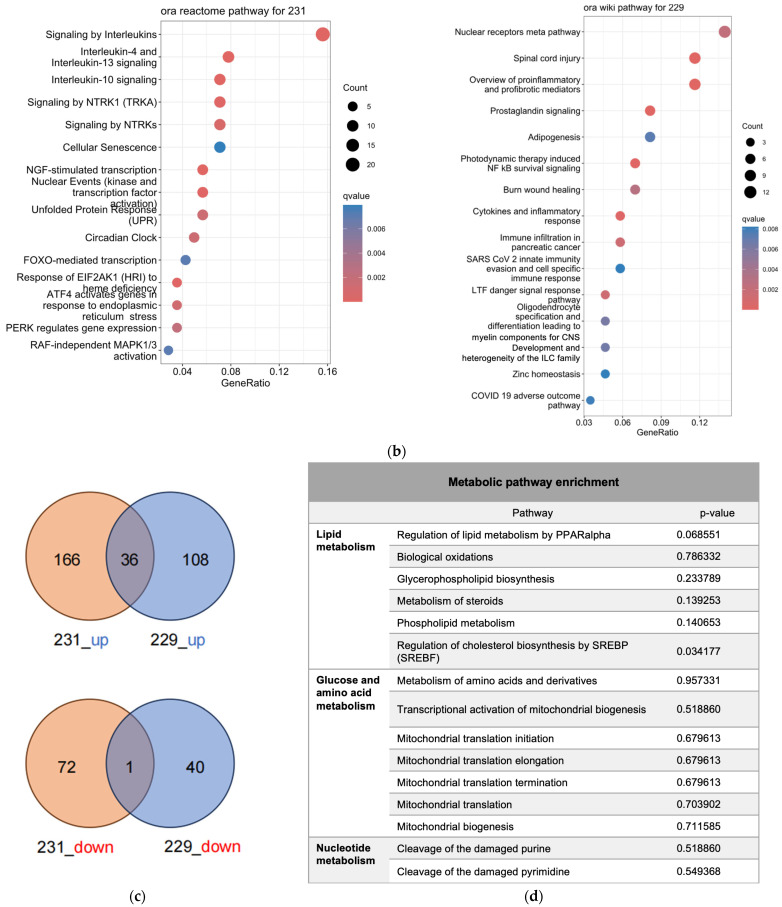
Transcriptomics analysis of MDA-MA-231 and LN229 cells. (**a**) KEGG pathway enrichment analysis of differentially expressed genes; (**b**) Volcano plots showing gene expression changes; (**c**) Venn diagrams of upregulated and downregulated genes across both cell types; (**d**) Common enriched pathways identified in both MDA-MB-231 and LN229 cells.

To minimize cell line-specific biases, transcriptomic analyses were conducted in two distinct cell lines, MDA-MB-231 and LN229. Cells were treated with 10 μM h-SPM, and untreated cells served as controls. Differential gene expression analysis revealed numerous upregulated and downregulated genes upon h-SPM treatment, as shown in the volcano plots (Figure 2a, Table S1). Genes with statistically significant changes were highlighted in red (upregulated) and blue (downregulated).

Bioinformatics analysis identified distinct sets of enriched pathways in each cell line (Figure 2b, Table S2). In MDA-MB-231 cells (reactome database), significantly enriched pathways included interleukin signaling (e.g., IL-4, IL-10, IL-13), cellular stress responses (e.g., senescence, unfolded protein response), and signaling cascades such as NTRK1, circadian rhythm, FOXMED-regulated transcription, and MAPK1/3 activation. In LN229 cells (wikipathway database), enriched pathways encompassed nuclear receptor signaling, inflammatory and immune responses, adipogenesis, NF-κB survival signaling, cytokine signaling, and pathways relevant to viral response and tissue repair.

Despite cell line-specific differences, pathways related to pro-inflammatory signaling and cellular stress were commonly enriched in both lines. To further investigate the overlap, Venn diagram analysis identified common differentially expressed genes, which were subjected to pathway enrichment (Figure 2c). Notably, several metabolic pathways—especially those involving amino acid, lipid, and mitochondrial metabolism—were significantly represented (Figure 2d), suggesting that h-SPM modulates cancer cell metabolism at the transcriptional level.

### 3.3. CE-MS Analysis Identifies h-SPM–Induced Changes in Polar Metabolites

**Figure 3:**
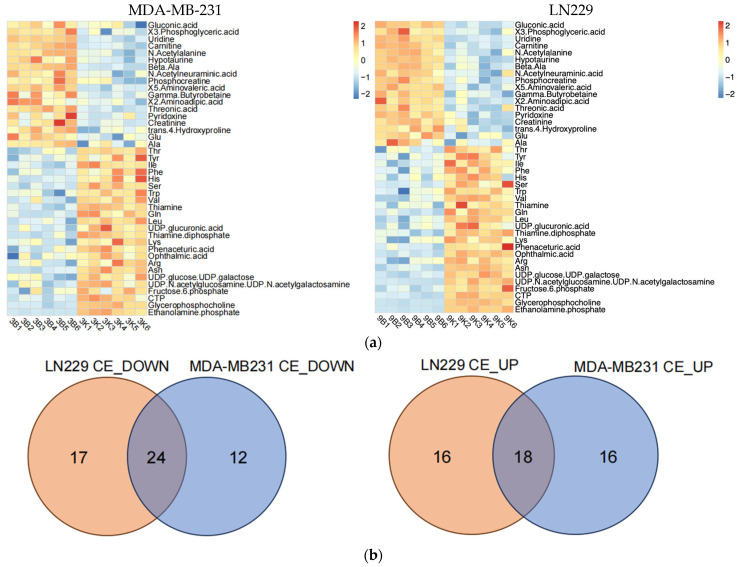

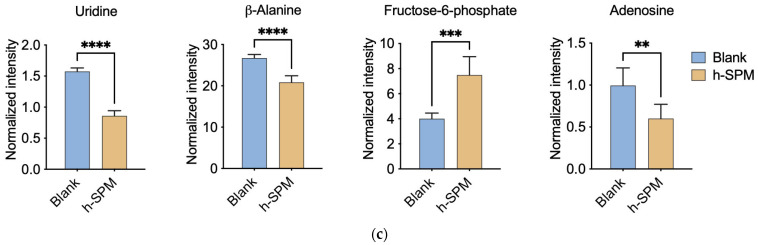
Metabolic alterations induced by h-SPM as detected by CE-MS. (**a**) Heatmap of significantly altered metabolites in MDA-MB-231 and LN229 cells (3: MDA-MB231; 9: LN229; B: blank; K: h-SPM treated. 1, 2, 3, 4, 5, 6: number of the replicative sample. For example, 3K3 represents the third replicative sample of MDA-MB231 cell treated with h-SPM). 3B1, representing treen duplication of MDA-MB231; (**b**) Venn diagrams of upregulated and downregulated metabolites; (**c**) Representative histograms of significantly changed metabolites. Each column represents the mean ± S.E.M. ** *p* < 0.01, *** *p* < 0.001, **** *p* < 0.001.

To precisely understand how h-SPM regulated cancer cell metabolism, we applied CE-MS and LC-MS. Firstly, CE-MS was employed to assess changes in polar metabolites in MDA-MB-231 and LN229 cells following h-SPM treatment. Heatmaps revealed distinct alterations in metabolite profiles between treated and control groups (Figure 3a, Table S3). A comparative analysis using a Venn diagram (Figure 3b) showed that 24 metabolites were consistently downregulated, and 18 were upregulated across both cell lines. Key metabolites are illustrated in bar charts (Figure 3c).

Following h-SPM treatment, intracellular levels of various amino acids were elevated. In addition, fructose-6-phosphate—a glycolytic intermediate—was upregulated. In contrast, metabolites such as adenosine, uridine, and β-alanine were decreased, implicating disruptions in glucose, amino acid, and nucleotide metabolism.

**Figure 4:**
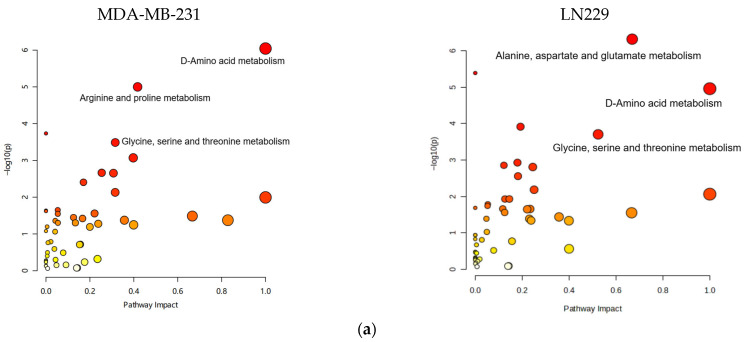

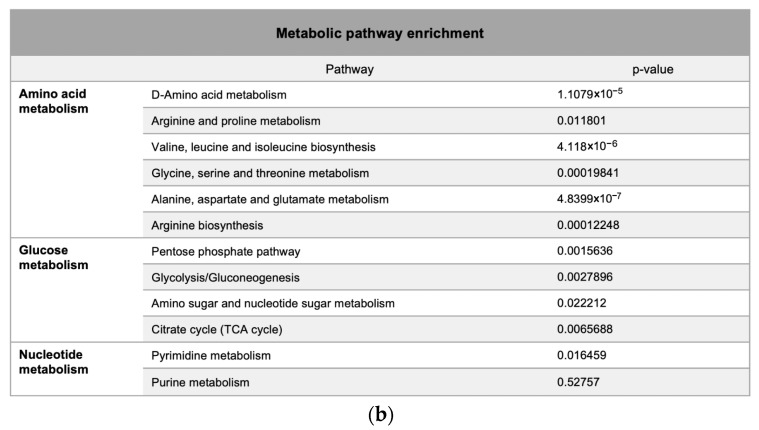
Pathway enrichment analysis based on CE-MS data. (**a**) Metabolite pathway enrichment analysis; (**b**) Common enriched pathways shared by MDA-MB-231 and LN229 cells.

Metabolic pathway mapping highlighted common pathways altered by h-SPM, including those involved in amino acid, glucose, and nucleotide metabolism (Figure 4a). A full list of affected pathways is shown in Figure 4b and Table S4. Integration with transcriptomic data further confirmed that h-SPM exerts broad regulatory effects on cellular metabolism.

### 3.4. Lipidomic Profiling Reveals Lipid Metabolism Alterations in h-SPM–Treated Cells

**Figure 5:**
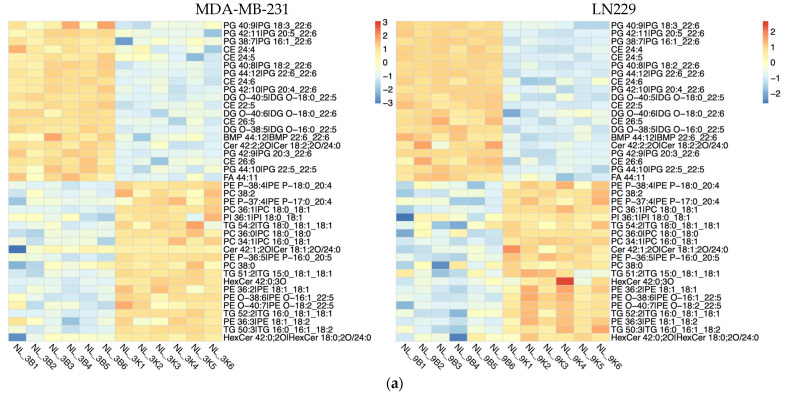

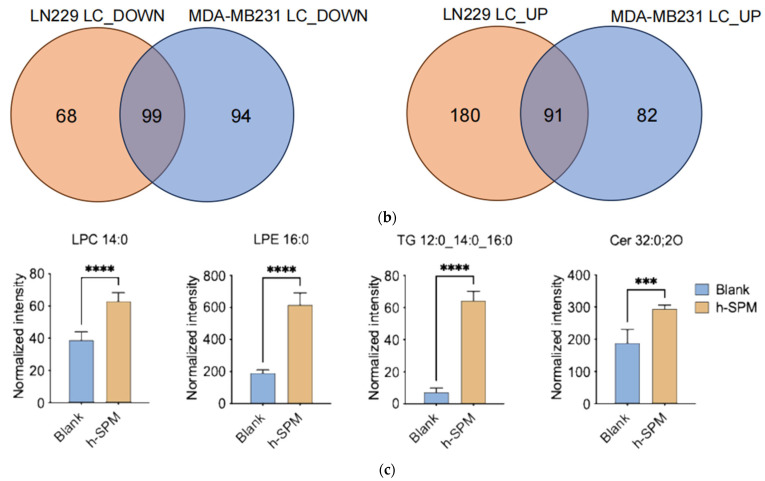
Lipidomic alterations induced by h-SPM as detected by LC-MS. (**a**) Heatmap of significantly altered lipids in MDA-MB-231 and LN229 cells (B: blank; K: h-SPM treated; NL: negative mode of liquid chromatography-mass spectrometry); (**b**) Venn diagrams of upregulated and downregulated lipids; (**c**) Representative histograms of significantly changed lipids. Each column represents the mean ± S.E.M. *** *p* < 0.001, **** *p* < 0.001.

Lipidomic analysis using LC-MS was conducted to evaluate the impact of h-SPM on lipid metabolism in MDA-MB-231 and LN229 cells. Heatmaps demonstrated distinct changes in lipid metabolite profiles post-treatment (Figure 5a, Table S5). Venn diagram analysis identified shared and cell line-specific lipid alterations. Among the downregulated lipid metabolites, 24 were common between both cell lines, while 17 and 12 were unique to LN229 and MDA-MB-231, respectively. Similarly, 18 upregulated lipid species were shared, with 16 unique to each line (Figure 5b).

Selected lipid metabolites showing significant changes are presented in Figure 5c. These include ceramide (Cer 32:0:20), lysophosphatidylcholine (LPC 14:0), lysophosphatidylethanolamine (LPE 16:0), and triglyceride (TG 12:0_14:0_16:0), suggesting that h-SPM exerts substantial effects on lipid homeostasis.

**Figure 6:**
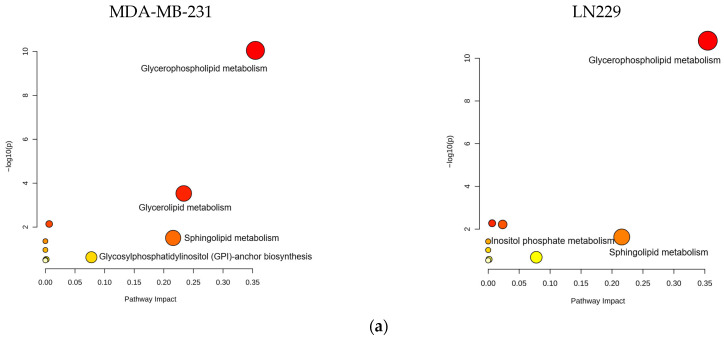

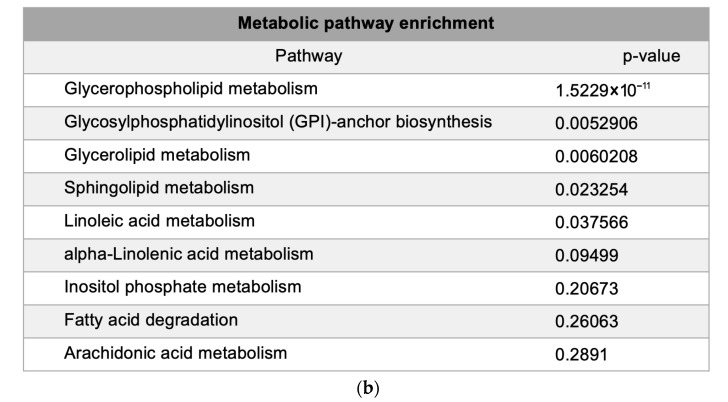
Pathway enrichment analysis of lipidomic data from LC-MS. (**a**) Enrichment. Analysis of lipid-associated metabolic pathways. (**b**) Shared lipid metabolic pathways altered in both cell types.

Bioinformatics analysis of the altered lipid metabolites mapped them to specific lipid metabolic pathways, including glycerophospholipid, glycerolipid, and sphingolipid metabolism (Figure 6a,b, Table S6). These pathways are strongly associated with the anticancer mechanisms of h-SPM.

### 3.5. h-SPM Downregulates NR1D1 Protein Expression

**Figure 7:**
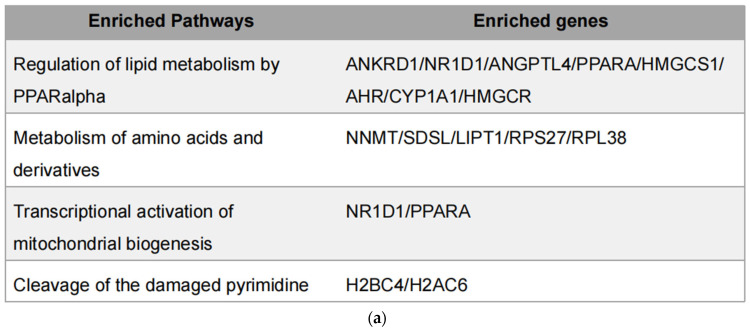

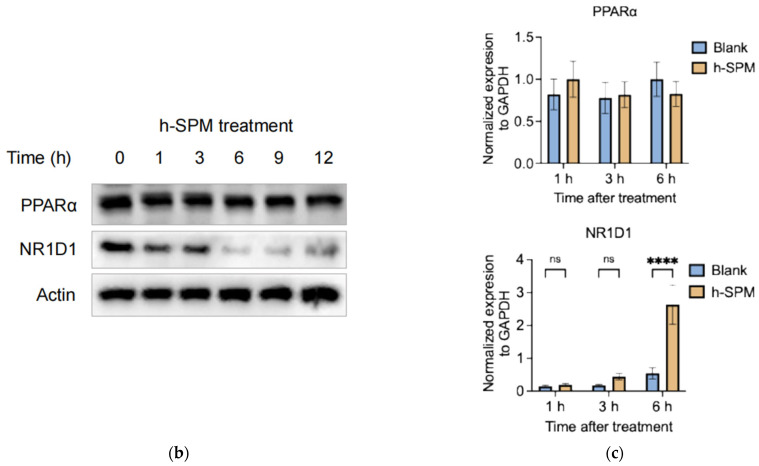
Integrated transcriptomic and metabolomic analysis. (**a**) Overlapping genes identified from combined transcriptomic and metabolomic datasets; (**b**) Protein levels of overlapping genes validated by Western blot in MDA-MB-231 cells; (**c**) mRNA expression of overlapping genes confirmed by qPCR in MDA-MB-231 cells. Each column represents the mean ± S.E.M. **** *p* < 0.001. The meaning of “ns” is “no significance”.

Integration of metabolomic and transcriptomic data identified four overlapping pathways (Figure 7a), with genes such as PPARα and NR1D1 appearing repeatedly. PPARα is a central regulator of lipid oxidation and energy metabolism [27,28], while NR1D1 plays roles in circadian regulation, inflammation, and metabolism as a heme-binding nuclear receptor that functions as a transcriptional repressor [29]. Through heme-dependent corepressor recruitment, NR1D1 represses transcription of key genes involved in lipid metabolism, gluconeogenesis, and inflammation [30]. Its dysregulation has been linked to metabolic disorders, making it a promising therapeutic target [31,32]. These two genes were prioritized for further experimental validation, since their established involvement in lipid metabolism and inflammation.

Western blot analysis showed that h-SPM treatment led to a time-dependent reduction in NR1D1 protein levels (Figure 7b), despite a corresponding increase in NR1D1 mRNA levels (Figure 7c). In contrast, PPARα expression remained unchanged at both the mRNA and protein levels. These findings suggest that h-SPM may modulate NR1D1 through post-transcriptional or post-translational mechanisms, implicating NR1D1 as a potential functional target of h-SPM.

### 3.6. h-SPM Induces ROS and Activates Pro-Inflammatory Signaling

ROS are key mediators of oxidative stress and play important roles in inflammation and metabolism [33]. Transcriptomic analysis indicated that h-SPM affects pathways associated with metabolism and inflammation, prompting investigation into its effect on ROS.

**Figure 8:**
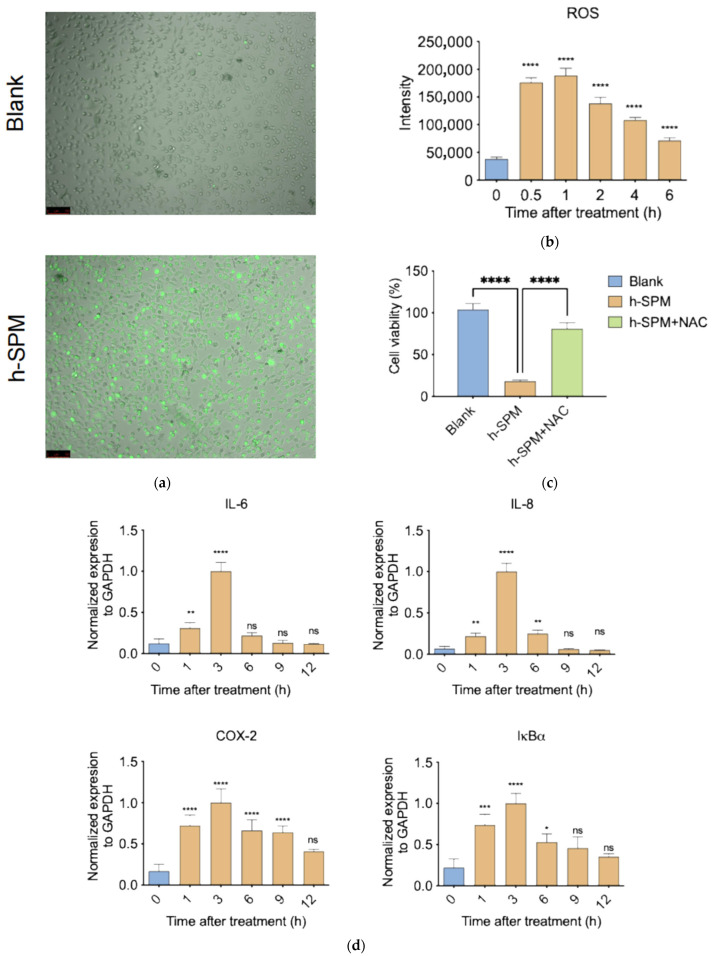
h-SPM-induced reactive oxygen species (ROS) accumulation and inflammatory response. (**a**) Detection of intracellular ROS in MDA-MB-231 cells using fluorescent staining; (**b**) Time-dependent increase in ROS levels under h-SPM treatment in MDA-MB-231 cells; (**c**) N-acetylcysteine (NAC) partially rescues h-SPM-induced cytotoxicity in MDA-MB-231 cells; (**d**) mRNA expression of inflammation-related genes analyzed by qPCR in MDA-MB-231 cells. Each column represents the mean ± S.E.M. * *p* < 0.05, ** *p* < 0.01, *** *p* < 0.001, **** *p* < 0.001. The meaning of “ns” is “no significance”. Scale bar: 100 μm.

ROS staining revealed a strong increase in intracellular ROS levels following h-SPM treatment, as indicated by enhanced green fluorescence (Figure 8a and Figure S1). Quantification showed a 4–5-fold increase in ROS within 30 min (Figure 8b), suggesting rapid induction of oxidative stress.

To assess the functional relevance of ROS, cells were co-treated with h-SPM and the ROS scavenger N-acetylcysteine (NAC) [34]. NAC co-treatment significantly restored cell viability, with survival rates comparable to control cells (Figure 8c), confirming that ROS generation is a major contributor to h-SPM–induced cytotoxicity.

Given the link between ROS and inflammation, we examined expression of pro-inflammatory genes following h-SPM exposure. qPCR analysis revealed a time-dependent increase in pro-inflammatory gene expression, peaking at 3 h post-treatment and declining thereafter (Figure 8d). This temporal pattern mirrors ROS dynamics, suggesting that h-SPM may induce inflammation via ROS-mediated signaling.

## 4. Discussion

In this study, we demonstrate the potent anticancer potential of h-SPM. Treatment with h-SPM at concentrations ranging from 3 to 10 μM significantly reduced cell viability. The rapid phosphorylation of p38 and cleavage of PARP indicated the induction of apoptosis following h-SPM exposure [25,26]. Further mechanistic investigation revealed that this apoptotic response was driven by an early and robust increase in intracellular ROS.

h-SPM is a synthetic derivative of a 16-membered macrolide antibiotic. Like other macrolides, it exhibits notable anticancer properties. While previous studies have described potential mechanisms underlying the anticancer effects of macrolides [35], the precise mode of action of h-SPM has remained unclear. To address this, we employed an integrative multi-omics approach, combining transcriptomic and metabolomic analyses to elucidate the compound’s biological activity.

Metabolomic profiling via CE-MS revealed substantial alterations in key metabolic pathways upon h-SPM treatment. Notably, polar metabolite analysis showed accumulation of glycolytic intermediates (e.g., fructose-6-phosphate, glucose-6-phosphate), a marked reduction in pyrimidine nucleotides (e.g., uracil), and elevated levels of multiple amino acids. This metabolic shift is indicative of enhanced glycolytic flux, likely reflecting mitochondrial dysfunction and a compensatory shift toward anaerobic metabolism [36]. In parallel, a pronounced decrease in β-alanine—a metabolite implicated in mitochondrial repair [37]—further supports the notion of h-SPM-induced mitochondrial damage and ROS generation [38].

Complementary lipidomic profiling revealed profound alterations in membrane lipid composition. LC-MS analysis identified elevated levels of ceramides (Cer), lysophosphatidylcholines (LPC), and lysophosphatidylethanolamines (LPE)—lipid species derived from the degradation of structural membrane components. These changes are consistent with widespread membrane remodeling. Ceramides, in particular, are well-established mediators of apoptosis [39,40], suggesting a role for membrane destabilization in h-SPM-induced cell death. Taken together, these findings support a dual mechanism of action: (1) disruption of mitochondrial function resulting in metabolic reprogramming and ROS accumulation, and (2) degradation of cellular membranes leading to the generation of pro-apoptotic lipid mediators.

Transcriptomic analysis further revealed enrichment of inflammatory signaling pathways, particularly those involving NF-κB and various interleukins [41,42]. This was validated by qPCR, which showed a rapid and transient upregulation of key inflammatory genes. Peak mRNA expression was observed at 3 h post-treatment, followed by a progressive decline. The temporal dynamics suggest that h-SPM elicits an acute inflammatory response that coincides with, and is likely driven by, early ROS accumulation and subsequent apoptotic signaling. When the drug acts on the cells, there is a phenomenon of accumulation of ceramides and ROS and reduction in NR1D1 protein levels. These three factors are very likely to interact with each other within the cells. The increase in ROS leads to damage of the membrane system, resulting in the accumulation of ceramides. After the damage of the membrane systems of organelles such as mitochondria, the content of NR1D1 will also decrease along with the damage of mitochondria, and at the same time, more ROS will be produced. This positive feedback regulation will accelerate the destruction of cell structure and promote cell apoptosis.

Integrated bioinformatics analysis of transcriptomic and metabolomic datasets identified four core pathways consistently altered by h-SPM treatment. Among these, NR1D1 emerged as critical regulator. NR1D1, a nuclear receptor involved in circadian regulation, lipid metabolism, and mitochondrial function [29,30], was significantly downregulated at the protein level, despite increased transcript expression. This phenomenon is quite unusual. The decrease in protein level is related to the damage of the associated cellular organelles, which led to the breakdown of this protein. However, the increase in mRNA level might be the negative feedback effect resulting from the decrease in protein level. The cells can self-rescue through this regulation while enduring damage. The downregulation of NR1D1 protein could be attributed to several post-transcriptional mechanisms. For instance, increased ROS levels are known to promote proteasomal activation and protein degradation. It is also plausible that h-SPM induces post-translational modifications, like ubiquitination, that target NR1D1 for degradation. Further studies are warranted to elucidate the exact mechanism underlying NR1D1 suppression. Furthermore, ROS measurements confirmed an early burst in ROS levels—peaking at 1hour post-treatment—preceding the upregulation of inflammatory markers. This sequential pattern supports a model in which mitochondrial impairment and ROS production act as upstream events that initiate inflammation and cell death.

## 5. Conclusions

In summary, h-SPM exerts potent anticancer effects through ROS generation, which resulted from the synergistic induction of mitochondrial dysfunction and metabolic dysregulation. Mitochondrial impairment leads to metabolic reprogramming toward glycolysis, depletion of β-alanine, a key metabolite in mitochondrial repair, and a rapid surge in ROS within 1 h of treatment. This oxidative burst activates an inflammatory cascade mediated by NF-κB and interleukins, peaking at 3 h. Simultaneously, the accumulation of pro-apoptotic ceramides occurs. Notably, suppression of NR1D1 protein level exacerbates metabolic stress by disrupting lipid homeostasis and mitochondrial regulation. The convergence of energy depletion, oxidative stress, inflammatory signaling, and ceramide accumulation possibly overwhelms cellular defenses, resulting in irreversible cell death.

## Data Availability

The RNA-seq and metabolomics data are available from the China National Center for Bioinformation (https://www.cncb.ac.cn/ (accessed on 7 July 2025)), the accession number of RNA-seq data is HRA012187 (https://ngdc.cncb.ac.cn/gsa-human/s/juuqU1hT (accessed on 7 July 2025)), the accession number of metabolomics data is OMIX010861 (https://ngdc.cncb.ac.cn/omix/preview/7pcBbLeY (accessed on 7 July 2025)).

## Supplementary Materials

The following supporting information can be downloaded at: https://www.mdpi.com/article/10.3390/metabo15100647/s1, Figure S1: The original images of ROS detection; Table S1: A full list of genes detected by RNA sequencing; Table S2: A full list of pathways obtained by transcriptomics analyses; Table S3: A full list of metabolites detected by CE-MS; Table S4: A full list of pathways obtained by CE-MS-based metabolomics analyses; Table S5: A full list of metabolites detected by LC-MS; Table S6: A full list of pathways obtained by LC-MS-based metabolomics analyses.

## Abbreviations

The following abbreviations are used in this manuscript:

h-SPM	n-hexyl spiramycin
CE-MS	Capillary electrophoresis–mass spectrometry
LC-MS	Liquid chromatography–mass spectrometry
ISP	Isovalerylspiramycin
DMEM	Dulbecco’s Modified Eagle Medium
FBS	Fetal bovine serum
CCK8	Cell counting kit-8
SDS-PAGE	Sodium dodecyl sulfate-polyacrylamide gel electrophoresis
UPLC	Ultra Performance Liquid Chromatography
TOF	Time of flight
ROS	Reactive oxygen species
NAC	N-acetylcysteine
