# Supplementary figures and images for: Uncovering Anticancer Mechanisms of Spiramycin Derivatives Using Transcriptomic and Metabolomic Analyses
Source: Metabolites. 2025 Sep 27;15(10):647. doi: 10.3390/metabo15100647 (PMC12566170; doi:10.3390/metabo15100647)

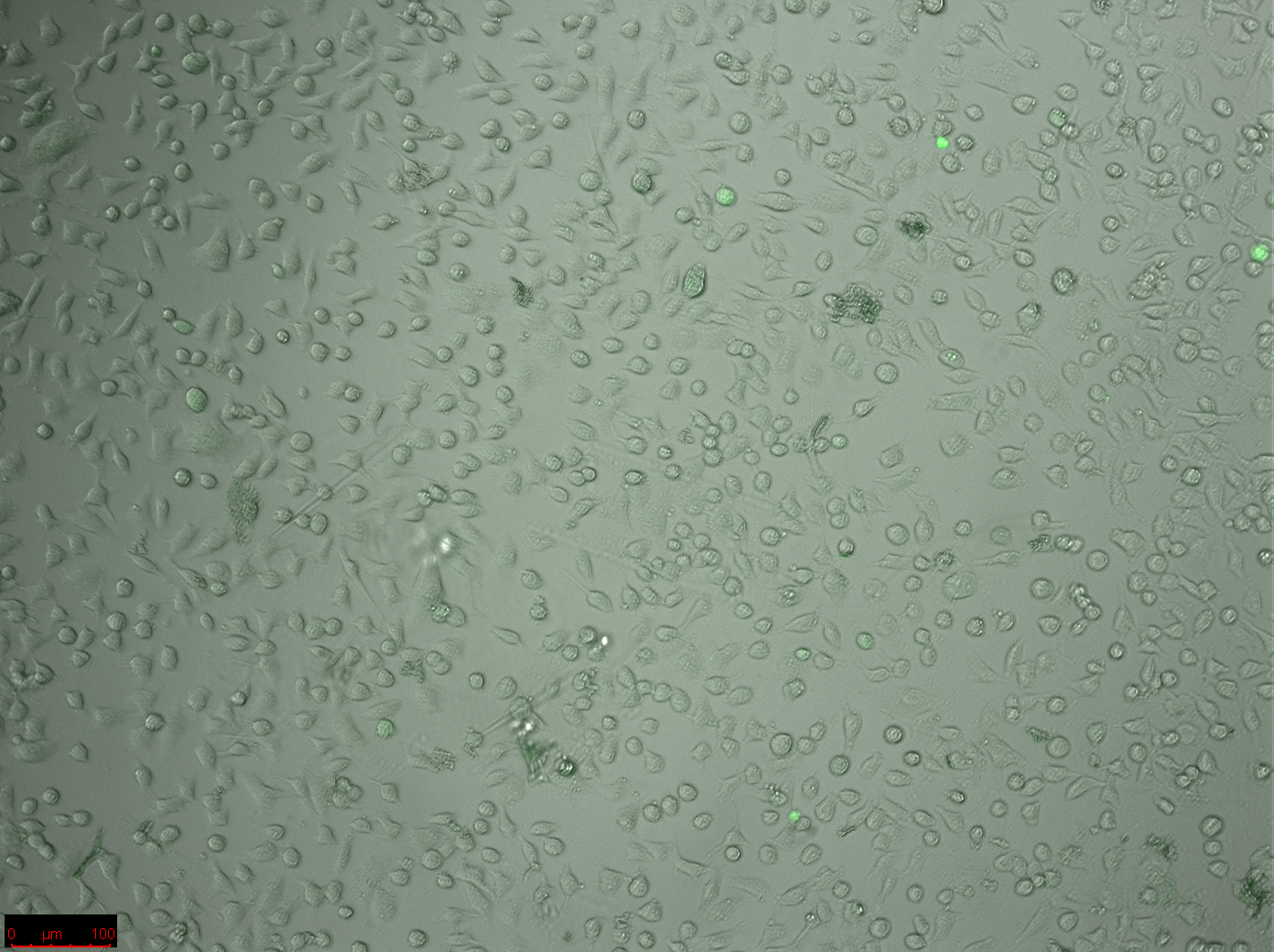

Supplement: Supplementary file 1 [file metabolites-15-00647-s001.zip › Figure S1 The original images of ROS detection/Project_blank.tif]

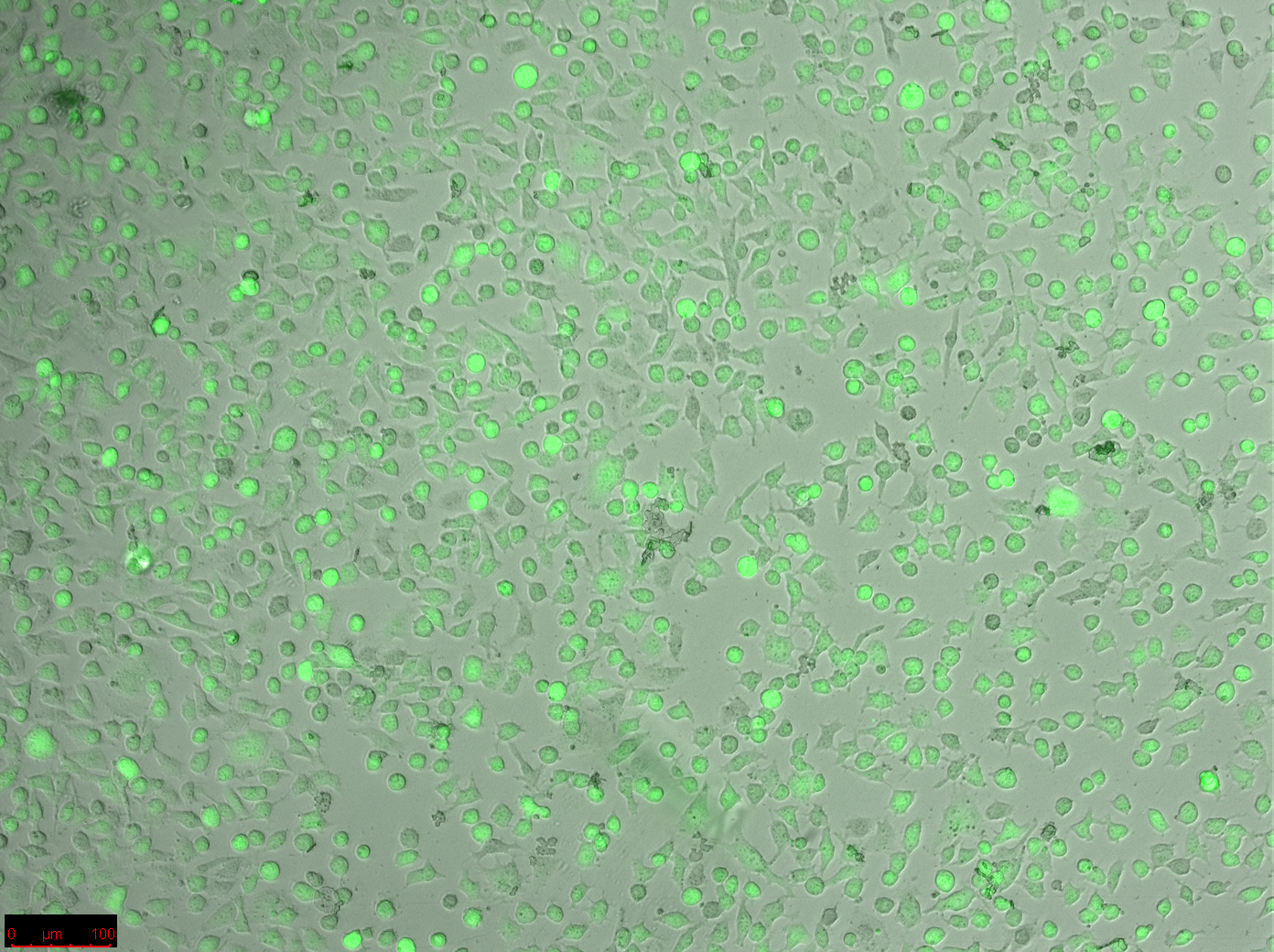

Supplement: Supplementary file 1 [file metabolites-15-00647-s001.zip › Figure S1 The original images of ROS detection/Project_drug.tif]
